# Therapeutic Potential of Inducible Endogenous Cytoprotective Heme Oxygenase-1 in Mitigating SARS-CoV-2 Infection and Associated Inflammation

**DOI:** 10.3390/antiox11040662

**Published:** 2022-03-30

**Authors:** Subhash Dhawan

**Affiliations:** Retired Senior FDA Research & Regulatory Scientist, 9890 Washingtonian Blvd., #703, Gaithersburg, MD 20878, USA; dr.subhash.dhawan@protonmail.com; Tel.: +1-(202)-731-9886

**Keywords:** heme oxygenase-1, hemin, viral infections, SARS-CoV-2, host defense, innate immunity

## Abstract

The inducible cytoprotective enzyme heme oxygenase-1 (HO-1) has gained significant recognition in recent years for mediating strong cellular resistance to a broad range of viral infections, regardless of the type of viruses, viral strains, or mutants. HO-1 is not a typical antiviral agent that targets any particular pathogen. It is a “viral tropism independent” endogenous host defense factor that upon induction provides general cellular protection against pathogens. By virtue of HO-1 being widely distributed intracellular enzyme in virtually every cell, this unique host factor presents a novel class of generic host defense system against a variety of viral infections. This Viewpoint proposes pharmacological evaluation of the HO-1-dependent cellular resistance for its potential in mitigating infections by deadly viruses, including the current severe acute respiratory syndrome coronavirus-2 (SARS-CoV-2), its variants, and mutants. HO-1-dependent cellular resistance against SARS-CoV-2 can complement current medical modalities for much effective control of the COVID-19 pandemic, especially with constantly emerging new viral variants and limited therapeutic options to treat SARS-CoV-2 infection and associated severe health consequences.

## 1. Introduction

Coronaviruses constitute a large group of viruses known to cause illnesses similar to common cold and more severe diseases, including severe acute respiratory syndrome (SARS) and Middle East respiratory syndrome (MERS). A novel class of coronavirus SARS-CoV-2 causes deadly severe upper respiratory illnesses, the coronavirus disease 2019 (COVID-19), and many serious secondary health complications was first identified in 2019 [1,2,3]. It is readily transmissible to others in close proximity and has become a clinical threat to the general population and healthcare workers worldwide. Mutations in the spike protein of SARS-CoV-2 present a moving difficult target for the development of variant-specific vaccines against this deadly virus. Consequently, the risk of new infections and potential spread of the disease due to recurrence of infections in the individuals who either recovered from COVID-19 or even those with administered vaccines are factual nightmares to control the pandemic. While immunization is a powerful and preferable medical preventive intervention against specific pathogens, an alternative or complementary approach is the activation of our own endogenous defense system has largely remained unaddressed. The self-defense innate mechanism acts immediately to resist all types of pathogens and retards rapid disease progression. It initially resists to the invading pathogens, the innate defense system. However, it is often rapidly taken over by infections with deadly viruses. Such failure of the immune system that protects the host against such pathogens, results in serious medical consequences, especially for deadly viral diseases such as COVID-19 caused by SARS-CoV-2, with limited medical options to effectively treat post-infection conditions. Rapidly emerging, new, highly contagious, and potentially more pathogenic SARS-CoV-2 variant strains and mutants continue to be difficult challenges to evaluate the effectiveness of vaccines against all emerging variant mutants. These concerns warrant a close evaluation of another crucial component of viral immunology and the first line of host defense mechanism, known as cellular immunity, against all invading pathogens, which has yet to be fully explored.

## 2. HO-1 in Host Defense against Viral Infections

**Figure 1:**
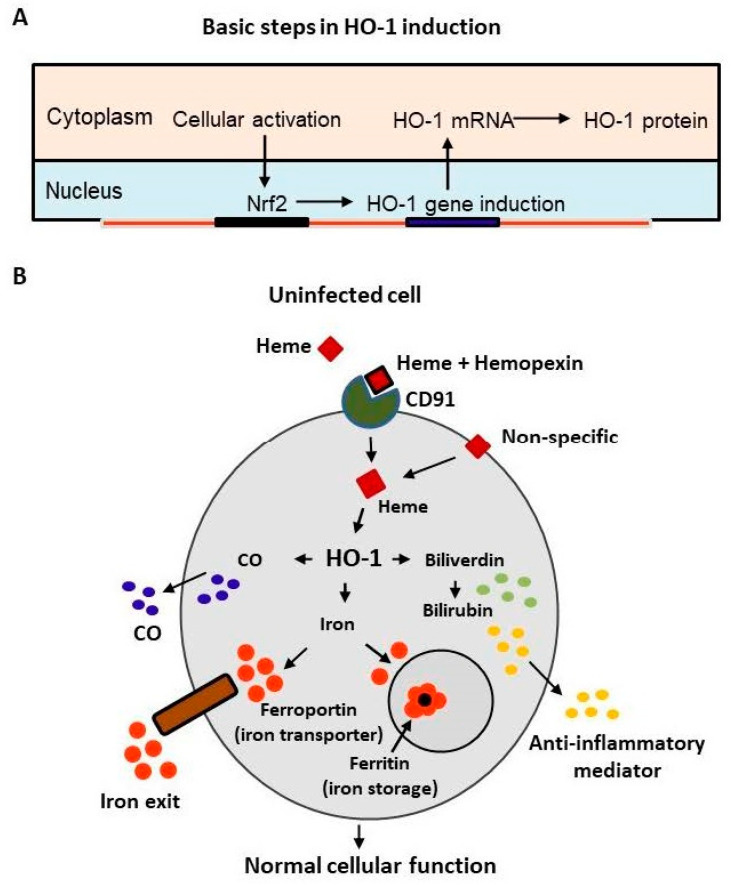
(**A**) Critical basic steps in the pathway of HO-1 regulation via its upstream transcription factor Nrf2 upon activation in response to extracellular signaling followed by translocation from nucleus to cytoplasm. (**B**) Diagram illustrating the involvement of HO-1 in iron homeostasis in normal cellular function, depicting HO-1-dependent hemoglobin and heme catabolic pathway. The activated HO-1 degrades hemoglobin and heme to biliverdin which is rapidly catalyzed by biliverdin reductase to bilirubin—a potent physiological mediator of the natural anti-inflammatory process.

HO-1 is a Nrf2-regulated gene that is activated by extracellular signaling and the HO-1 mRNA is translocated from nucleus to cytoplasm where it is translated into the functional HO-1 protein (Figure 1A). The inducible isoform of HO isozyme, HO-1, expressed at low basal levels in most cells and tissues [4], is markedly upregulated by its natural substrate heme leading to the generation of biliverdin, carbon monoxide, and release of ferrous iron as shown in Figure 1B. The activated HO-1 degrades heme to biliverdin which is rapidly catalyzed by biliverdin reductase to bilirubin—a potent physiological mediator of the natural anti-inflammatory process. Heme catabolized bilirubin functions as an endogenous modulator of inflammation, primarily by disrupting adhesion molecule-mediated leukocyte migration [5]. The objective of this article is to draw our attention specifically on HO-1 as a promising defense mechanism against SARS-CoV-2, based on several convincing published reports on cellular protection against a broad range of pathogens [6,7,8,9,10,11,12]. These compelling beneficial natural anti-viral outcomes by activated cellular HO-1 presents this inducible enzyme as a unique generic host defense system against viruses. Both heme and hemin are iron-containing porphyrin molecules that exhibit the same biological function. The only difference between heme and hemin is that heme contains ferrous cation (Fe^++^), whereas hemin contains ferric cation (Fe^+++^). In pharmacological frameworks, hemin is formulated with human albumin (e.g., hemin-drug Panhematin^®^) to reduce the risk of phlebitis (or Venitis) and to enhance the stability of the active compound.

## 3. Potential of HO-1 in Enhancing Cellular Resistance to SARS-CoV-2

Since the discovery of HO-1 in 1968 [13] as the crucial enzyme in scavenging the hemoglobin and heme toxicity effects by catalyzing pro-inflammatory hemoglobin and heme to their anti-inflammatory catabolic products bilirubin and CO [14], a substantial progress has been made to explore its potential against numerous pathological conditions, including viral infections. Heme is known to convert organic hydroperoxides into highly reactive alkoxyl radicals and promoting generation of toxic reactive oxygen species [Reviewed in reference [15]. However, being a natural substrate for HO-1, it is catabolized by induced intracellular HO-1 into non-toxic anti-inflammatory molecules, bilirubin and CO. There are numerous publications in recent years supporting the therapeutic utility of HO-1 induction against various pathogenic infections, first reported for HIV-1 in 2006 [6], and, now more recently, as a potentially viable therapeutic option for mitigating COVID-19 [16,17,18,19]. However, a recent study by Maestro et al. suggested that HO-1 induced by hemin could not inhibit SARS-CoV-2 infection [20]. These authors demonstrated the unchanged viral RNA levels from the cell culture supernatants, whereas Kim et al. [18] determined reduction in both viral RNA and viral protein in the cells after treatment with hemin. Kim et al. explained the contradictory results between their studies and Maestro et al. might be due to different experimental conditions and methods. While several agents efficiently induce HO-1 [21,22], hemin is particularly important because it is the active component of a previously FDA-approved drug for the treatment of acute porphyria with thus far no known serious adverse effects. In addition, hemin serves as a natural substrate for HO-1 that provides a strong innate host protection against pathogens, regardless of types, mutant strains of the pathogens, and even mycoplasma [12]. Collectively, these studies warrant the evaluation of hemin in clinical studies for its therapeutic potential against SARS-CoV-2 infections, particularly in situ, using a simple therapeutic swab device system comprising multifunctional drugs, directly to nasopharynx and posterior pharynx, the primary sites of upper respiratory infections, including SARS-CoV-2 [23].

An effective protection during early stage of viral exposure by restoring the viral-damaged host response is critical for our first defense against upper respiratory infections. In almost all viral infections, both pathogen and cellular gene transcription factors cooperatively promote active replication of the invaded pathogens. These factors contribute substantially from pathogen binding to the target cells and then internalization with subsequent replication, leading to the development of clinical symptoms followed by progression of the disease. HO-1 induction remarkably restricts replication of numerous viruses and thus is considered as a key mediator of the host defense mechanism.

Interaction between viruses and host cell regulation is a key component in the initial response to infections and subsequent pathogenic events. Therefore, overcoming the viral-host defense evasion mechanism is pivotal to stimulate innate cellular immunity. Physiologically stimulated cellular HO-1, a widely distributed, inducible cytoprotective enzyme, presents a unique opportunity to enhance the normal host cell protective response. Failure of the immune system to defend against an invading pathogen poses serious challenges in managing the medical consequences [24,25], particularly for diseases with limited options for effective post-infection treatment. Under these circumstances, induction of cellular defense responses would provide an alternative or concurrent therapeutic strategy for treating infections. The HO-1-dependent enhanced cellular resistance can be expected to complement current medical modalities for much effective control of the COVID-19 pandemic, especially in the absence of broadly protective vaccines and the limited choice of therapeutic options to treat post-infection conditions and associated severe secondary complications.

**Figure 2:**
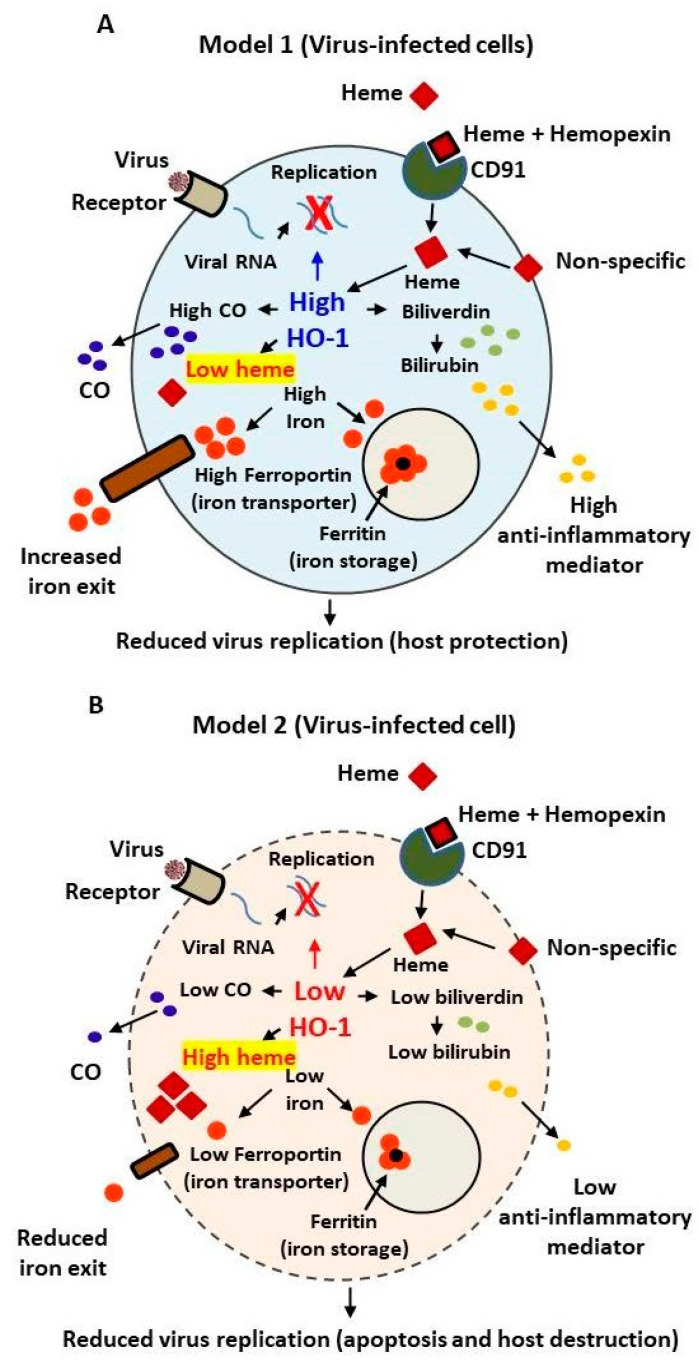
Models depicting the involvement of HO-1-dependent innate cellular resistance to viruses by reducing virus replication and host protection (**A**) or host destruction by heme cytotoxicity (**B**).

Stimulation of the hemin-induced HO-1-dependent defense system along with mass vaccination might also facilitate immunity against constantly emerging pathogens than vaccines alone. This can be accomplished by substantially reducing intracellular viral loads and/or possibly by selective heme-induced toxicity of less HO-1-expressing SARS-infected cells. Because of high affinity of heme for albumin and hemopexin, it is not toxic to normal cells. Figure 2 presents two speculative models for HO-1-dependent cellular response in a virus-infected cell. Based on HO-1-dependent inhibition of various virus replication, two possible scenarios can be expected: (a) one in which HO-1 is induced by heme, interfering with the virus–host protein interaction, required for active virus transcription, thus causing suppression of virus replication, yet simultaneously providing host protection by maintaining normal iron homeostasis as shown in Figure 2A; or (b) in which viral infection targets and reduces the basal HO-1 expression in virus-infected cells thereby low induction of HO-1 in response to heme. Such defective iron homeostasis in the infected cells may cause excessive intracellular iron accumulation causing heme cytotoxicity as shown in Figure 2B. The latter scenario is somewhat similar to that recently presented by Wagener et al. [26] who suggested an increased intracellular accumulation of iron in SARS-CoV-2 cells as a possibility of heme cytotoxicity in the infected cells. Nonetheless, in either case, virus production is expected to reduce with or without cellular protection. The reduced HO-1 expression in SARS-CoV-2-infected cells isolated from COVID-19 patients [17] that has recently been reported, supports the concept presented in Figure 2B.

Interventions involving activation of the powerful cellular HO-1-dependent cellular response by its natural substrate hemin could enable additional a safe viable option for reducing the severity of infections and disease progression. Therefore, HO-1-depenent strong resistance to various viruses for blocking or limiting their replication and associated pathogenesis should be explored to gain additional insights for mitigating SARS-CoV-2 infection. Scientific studies on evaluating the role of inducing HO-1 should provide an effective and productive outcome in advancing new therapeutic options.

Modulation of the innate HO-1 may pave ways to provide new key insights into disrupting the replication cycle between the viruses and host factors for an alternative or concurrent effective physiological therapeutic strategy to effectively ameliorate infections by SARS-CoV-2 or other emerging viral pathogens. Activation of cellular HO-1-dependent cellular response could enable additional safe viable option for reducing SARS-CoV-2 infections, especially in the early stage of infection. In addition, since the activation of the innate immune activation also triggers antigen-specific adaptive immune responses [27], HO-1 activation could potentially enhance the effectiveness of the secondary adaptive or humoral immune response produced naturally in recovered COVID-19 patients as well as in vaccine administered individuals.

Although the approach for stimulated innate HO-1-dependent cellular protection presented in this Viewpoint can be generally applicable to most viral infections due to its capacity to enhance strong cellular resistance, it is particularly pertinent to the current problems of constantly emerging SARS-CoV-2 viral mutants. A number of identified SARS-CoV-2 variants are associated with reduced capacity of neutralization by antibodies generated against individual viral strains either naturally or produced by vaccination, in addition to increased transmissibility and pathogenesis [1,2,3]. These serious concerns validate evaluation of the potential of the stimulated immune defense response along with continued vaccinations. Activation of innate cellular HO-1 in host defense may provide other therapeutic possibilities to address some of these important issues.

## 4. Potential of HO-1 in Regulating Innate Immune Function against SARS-CoV-2—Why Is it Critical for Managing COVID-19 Pandemic?

Innate cellular immunity is the first line of host defense mechanism against invading pathogens. The innate immune system plays an initial critical role in the clearance of viral infections. Furthermore, while the innate immune response is immediate, the secondary immune response is not generated until several days or weeks after infection. However, despite the powerful capacity of the initial innate host defense mechanism to resist all invading pathogens, it has not yet been adequately investigated as a form of initial protection against deadly viruses, such as SARS-CoV-2. Because HO-1 is widely distributed as an inducible intracellular enzyme in all cells, it presents a novel class of innate host defense system against deadly viruses. Therefore, pharmacological evaluation of HO-1-dependent strong cellular immunity may provide valuable insights into mitigating deadly viral infections, including SARS-CoV-2 mutant variants, and also for reducing their associated serious pathogenic consequences in a safe and effective manner. Physiologically induced innate HO-1 might also help facilitate the development of immunity against constantly emerging pathogens than vaccines alone by reducing viral loads and hence minimizing transmissions. Interventions involving activation of cellular HO-1-dependent cellular response could enable potentially safe viable options for reducing the severity of SARS-CoV-2 infections.

**Figure 3:**
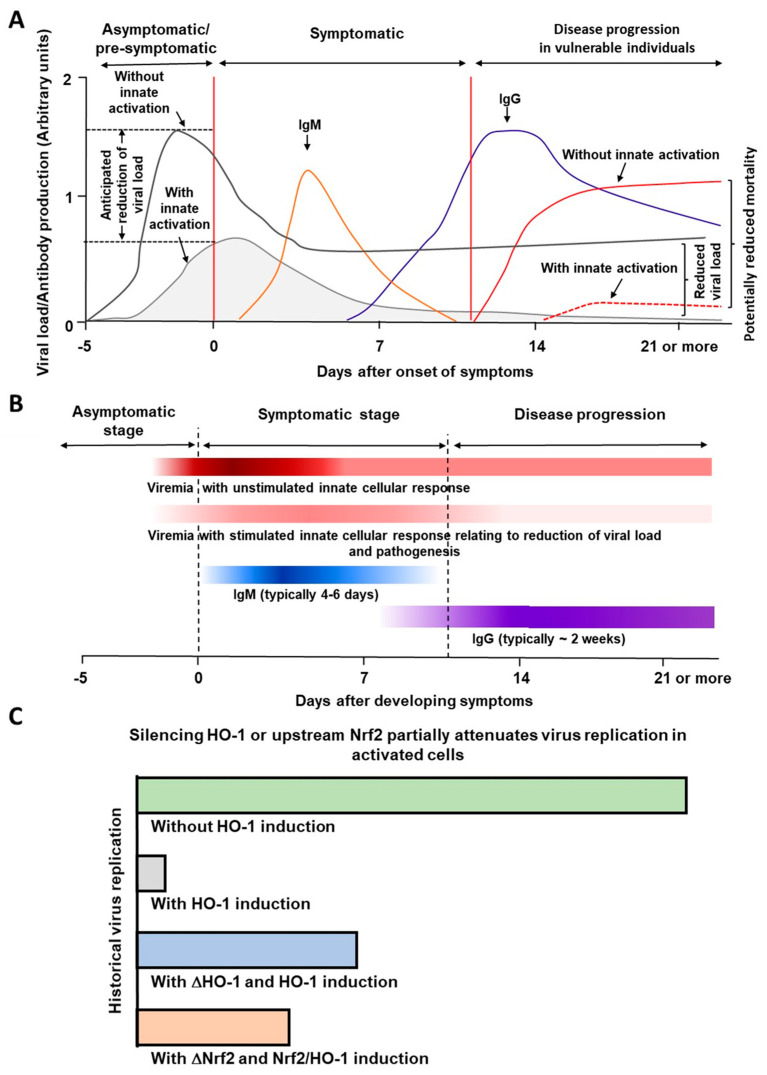
(**A**) A model depicting the importance of inducible innate cellular protective response against SARS-CoV-2 infection. (**B**) This model presents the concept of the stimulated innate immunity for reducing the viral load, retarding disease progression, and generation of the secondary humoral immune response. (**C**) A diagram illustrating the involvement of HO-1 and its upstream gene Nrf2 in reducing virus replication based on historical published data [8,9,12,17]. Panel A and Panel B are reproduced with permission from Refs. [28,29] Copyright 2021 Research Trends.

Figure 3A,B illustrate a graphical conceptualization of the potential benefit of rapidly inducing (or stimulating) the innate cellular response to reduce viral load and to retard disease progression in addition to generating the secondary immune responses. The typical patterns of viremia and subsequent generation of IgM and IgG responses shown in these diagrams are similar to those typically seen in most viral infections. As depicted in this diagram, activation of the innate host defense system might provide substantial reduction in virus replication in the cells of early or mildly symptomatic infected patients [28,29]. Consistent with these notions, a recent study has reported heme as an inducer for innate immune memory [30]. HO-1-dependent innate immune responses could potentially be induced by non-toxic treatment with hemin. Activating the innate host defense system could substantially reduce virus replication in early or mildly symptomatic COVID-19 patients to maintain an extremely low level of SARS-CoV-2. Figure 3C represents a model general pattern for HO-1-dependent inhibition of virus replication based on published historical observations [8,9,12,17].

Because COVID-19 is related to a marked dysregulation of the immunity by viral invasion of the innate immune system [31], emphasis needs to be given to evaluate the power of HO-1 to restore the cellular response against SARS-CoV-2. The induction of the host response to boost the initial cellular response to resist replication of the invading pathogens is expected to reduce the viral load and potentially retard the progression of the disease. In addition, it could also enable the generation of effective secondary immune response in the infected individuals to facilitate recovery. The physiological stimulation of the innate HO-1 along with the continued vaccination program may yield beneficial outcomes for controlling the COVID-19 pandemic. Since viruses utilize host factors for replication in infected cells and alter the immune system, directly or indirectly during inflammatory responses, HO-1 regulation of the innate immune response may provide promising additional therapeutic approaches to reduce the severity of SARS-CoV-2 infections. Such studies could enable critical insights into the innate immune response to SARS-CoV-2.

COVID-19 is an important area of an enormous current global public health concern. The HO-1-depenent resistance to viruses, viral replication and associated pathogenesis may provide additional medical modalities for SARS-CoV-2 that at present are of the highest priority. Therefore, future studies should also focus on the HO-1 regulatory pathways as viable therapeutic options for SARS-CoV-2 infections and in regulating innate host cellular response. Such studies, as stated above, could potentially enhance the effectiveness of the humoral immune response produced naturally in COVID-19 recovered patients or possibly in vaccines administered individuals.

## 5. Anti-Inflammatory Potential of HO-1 in COVID-19-Associated Clinical Consequences

The host immune response to the SARS-CoV-2 causes an excessive inflammatory reaction called cytokine storm. The cytokine storm in many COVID-19 cases is associated with tissue damages. Most commonly organs that are largely affected by SARS-CoV-2 infection is the lung. In most cases, the lung dysfunction is short-term, causing acute respiratory distress syndrome [32,33]. However, in more severe cases, the damage can persist for an extended period of time; especially, in individuals with chronic lung disease that are at a greater risk for severe COVID-19 and developing much severe pulmonary disorders. Due to insufficient oxygen supply to organs, among other elements, the hyperinflammatory conditions associated with SARS-CoV-2 infection can contribute to heart, lung, and other tissue damages [34].

Although, high level of inflammation called multisystem inflammatory syndrome is a rare but serious condition associated with COVID-19, which not just affects the heart and lungs, but other body organs, including kidneys, brain, eyes, or gastrointestinal organs are also inflamed [35]. These inflammatory conditions can be controlled by limiting the SARS-CoV-2 infections at the early stage. The innate cellular HO-1 activation is known for its role not only against oxidative stress and apoptosis, but also in mediating in potent anti-inflammatory functions [26] (and references therein), [36]. Since HO-1-dependent catabolic products bilirubin, biliverdin and CO exhibit potent anti-inflammatory functions, targeted pharmacological modulation of HO-1 should be taken into consideration for clinical manifestation of SARS-CoV-2 infection and effectively managing its associated inflammatory complications.

## 6. Summary and Perspectives

COVID-19 is an enormous current global public health concern. The concept of HO-1-depenent strong cellular resistance to a wide range of viruses should be considered to explore additional therapeutic options in managing SARS-CoV-2 infection. Because of its generic, non-specific protective role against several viral infections, regardless of the types of virus strain and mutant variants, pharmacological stimulation of HO-1 presents a potentially promising approach for SARS-CoV-2. Scientific studies on evaluating the role of HO-1 against SARS-CoV-2 may be expected to yield an effective and productive outcome in advancing new medical modalities, also as an alternative or concurrent therapeutic strategy for treating SARS-CoV-2 infections. Based on HO-1-dependent inhibition of numerous pathogens by hemin, the active component of an FDA-approved drug for an unrelated medical condition, it may further advance its therapeutic potential for mitigating SARS-CoV-2 infection and its associated inflammatory consequences with little or no adverse effects.
